# Encapsulation of *Annona muricata* Acetogenins in Self-Nanoemulsifying Drug Delivery Systems (SNEDDSs): Controlled Release and Antiproliferative Activity in MC-38 Colon Cancer Cells

**DOI:** 10.3390/life16101726

**Published:** 2026-10-15

**Authors:** Rubén R. Córdova-Espíritu, Diego A. Bravo-Alfaro, Arturo Mendoza-Galván, Hugo S. García, Gabriela Aguilar-Hernández, Efigenia Montalvo-González, Dulce M. Palmerín-Carreño, Christian Hernández-Guzman, Misael Rios-Ortiz, Teresa García-Gasca, Heber U. Pérez-Ortega, Gabriel Luna-Bárcenas, Francisco Villaseñor-Ortega

**Affiliations:** 1 Department of Biochemical and Environmental Engineering, Tecnológico Nacional de México en Celaya, Antonio García Cubas #600 esq. Av. Tecnológico, Celaya 38010, Guanajuato, Mexico; d2103017@itcelaya.edu.mx (R.R.C.-E.);; 2 Artificial Intelligence for Manufacturing and Supply Chain Institute and The School of Engineering and Sciences, Tecnológico de Monterrey, Queretaro 76130, Mexico; 3 Cinvestav Unidad Querétaro, Queretaro 76230, Mexico; amendoza@cinvestav.mx; 4 Unidad de Investigación y Desarrollo de Alimentos, Tecnológico Nacional de México/ Instituto Tecnológico de Veracruz, M.A. de Quevedo #2779, Col. Formando Hogar, Veracruz 91897, Mexico; 5 Comprehensive Food Research Laboratory, Tecnológico Nacional de México-Instituto Tecnológico de Tepic. Av. Tecnológico #2595 Fraccionamiento Lagos del Country, Tepic 63175, Nayarit, Mexico; 6 Chemistry Faculty, Universidad Autónoma de Querétaro, Campus Pedro Escobedo, Avenida Panamericana No. 180 Int. 100, Centro, Pedro Escobedo 76700, Queretaro, Mexico; dulce.palmerin@uaq.mx (D.M.P.-C.); christian.hernandezg@uaq.mx (C.H.-G.); 7 Laboratory of Cell and Molecular Biology, Faculty of Natural Sciences, Autonomous University of Queretaro, Campus Juriquilla, Av. De las Ciencias S/N, Queretaro 76230, Mexico

**Keywords:** acetogenins, SNEDDS, nanoemulsions, controlled release, in vitro digestion

## Abstract

Acetogenins (ACGs) are bioactive compounds typically found in the Annonaceae family, such as soursop (*Annona muricata*), and have demonstrated strong antineoplastic effects at very low doses across different cancer cell lines. However, their poor water solubility limits their bioavailability and bioactivity. This research aimed to prepare and evaluate self-nanoemulsifying drug delivery systems (SNEDDSs) to improve the solubility and efficacy of ACGs against colon cancer cells. The results indicated that ACGs could be effectively dissolved in medium-chain triglycerides, mainly composed of caprylic acid, reaching a concentration of 20 mg/mL. Pseudoternary phase diagrams were used to identify spontaneous nanoemulsification zones using Cremophor^®^ EL/Tween 80 as surfactants and polyethylene glycol as a cosurfactant. The resulting nanoemulsions exhibited droplet sizes ranging from 14.6 to 50.18 nm and polydispersity indices between 0.04 and 0.18. In vitro tests simulating gastrointestinal conditions showed the stability of nanoemulsion particle size for 105 min in a simulated small intestine environment. An in vitro release assay revealed a 92.2% release of ACGs incorporated into SNEDDSs after 7 h. Finally, cell-based assays demonstrated that ACG-loaded nanoemulsions exerted cytotoxic effects on MC-38 colon cancer cells, with an IC_50_ of 16.32 μg/mL. These findings support the potential of SNEDDS-encapsulated ACGs as an effective strategy for colon cancer therapy.

## 1. Introduction

Oral drug administration is popular among patients because it is noninvasive and easy to self-administer [1]. However, many drugs have poor water solubility, low bioavailability and limited therapeutic effectiveness, as the body struggles to absorb and deliver them to their target sites. This issue also affects acetogenins (ACGs), a group of polyketides with 35–37 carbon atoms that contain one to three tetrahydrofuran rings and one or two β,γ-lactone rings. These compounds are mainly extracted from Annonaceae species, such as soursop (*Annona muricata*). ACGs exhibit multiple biological activities, especially antineoplastic [2], antibacterial [3], and antifungal ones [4]. Their anticancer potential is particularly important, as several in vitro studies have shown that ACGs can inhibit the growth of prostate, breast, lung, pancreatic, and colon cancer cells. Notably, colon cancer is the third most diagnosed cancer worldwide and the second leading cause of cancer-related death, with an estimated 1.14 million new cases and 0.538 million deaths in 2022 [5].

Nanotechnology-based delivery systems can greatly improve the bioavailability of ACGs [6]. Because of their highly lipophilic nature, ACGs have very low aqueous solubility (below 1 μg/mL) [7], which results in minimal gastrointestinal absorption [8]. Various drug delivery methods, such as polymeric particles, nanosuspensions, and nanoemulsions, have been created. Recent examples include nanosuspensions and functionalized poly(glycerol adipate) nanoparticles that notably boost antitumor activity [9,10].

Self-nanoemulsifying drug delivery systems (SNEDDSs) represent an appealing approach to orally delivering lipophilic bioactives. SNEDDSs are uniform mixtures of oil, a surfactant, and a cosurfactant that automatically form nanoemulsions when in contact with aqueous media under gentle agitation, simulating gastrointestinal conditions. These systems have consistently demonstrated the ability to enhance the solubility and oral bioavailability of poorly water-soluble compounds [11]. Their novel formulation makes them especially suitable for ACG delivery.

Previous studies have used crude Annona extracts in various nanocarriers, as encapsulating an acetogenin-enriched fraction offers better dose accuracy, allows for the evaluation of molecule-specific activity, and aids in optimizing pharmacokinetic and toxicological profiles. Recent advancements in nanoencapsulated ACGs have shown improved cellular uptake, sustained release, and increased cytotoxic potency against tumor cell lines, emphasizing the importance of ongoing research into diverse nanocarrier platforms, including nanosuspensions, functionalized polymeric nanoparticles, and, notably, SNEDDSs, to enhance therapeutic efficacy while reducing adverse effects [9,10,11].

The objective of this work was to use an ACG-enriched fraction to develop a nanosystem offering a potential therapeutic alternative for cancer, specifically colon cancer, by nanoencapsulating ACGs into an optimized SNEDDS. Accordingly, we aimed to design and characterize a SNEDDS capable of incorporating ACGs, assess its suitability for oral administration, and evaluate its in vitro cytotoxicity against colon cancer cells.

## 2. Materials and Methods

### 2.1. Materials

The ACG samples, extracted from *Annona muricata* seeds, were kindly supplied by the Comprehensive Food Research Laboratory, Laboratorio Integral de Investigación en Alimentos at the Tecnológico Nacional de México, Tepic. They were obtained through the sonication extraction of the seeds, following a previously reported method [12]. Cremophor^®^ EL, medium-chain triglycerides (MCTs), Tween 80, polyethylene glycol, porcine mucin, α-amylase, pancreatic lipases, and porcine pepsin, along with reagents for Kedde’s reagent preparation, such as 3,5-dinitrobenzoic acid, potassium hydroxide, and absolute-grade methanol, were purchased from Sigma-Aldrich (St. Louis, MO, USA).

The salts used in the in vitro gastrointestinal studies, such as sodium chloride, ammonium nitrate, hydrochloric acid, potassium phosphate, potassium chloride, potassium citrate, urea, and calcium chloride, were obtained from J.T. Baker (Mexico City, Mexico).

### 2.2. Solubilization of ACGs in MCT as Oil Phase

An oil phase solubility study of ACGs was performed following Sulkhan et al. [13], with minor modifications. A total of 20 mg of ACGs was dissolved in 1 mL of MCT and stirred on an orbital shaker for 20 min at a constant temperature of 50 °C. The samples were stored at room temperature for later use.

### 2.3. Quantification of Acetogenins by UV-VIS Spectroscopy

Kedde’s reagent was used, which reacts with α, β-unsaturated or saturated γ-lactone rings to produce a pink color, as described by Aguilar-Hernández et al. [12]. Because Kedde’s reagent reacts with the lactone moiety characteristic of ACGs, this assay provides a non-specific estimation of the total lactone-bearing acetogenin content rather than the concentration of individual compounds. Accordingly, the reported values are expressed as total ACG equivalents (ACG equivalents) and should not be interpreted as the concentration of any single ACG. Specifically, 200 μL of each sample was mixed with 200 μL of Kedde’s reagent, and absorbance was measured at 505 nm using a UV-VIS spectrophotometer (Jenway 6705, Dunmow, UK). The results were expressed as milligrams of ACGs per gram of formulation (mg/g).

### 2.4. SNEDDS Formulation

SNEDDSs were prepared following [14], with minor modifications. Medium-chain triglycerides (MCTs) were used as the oil phase in volumes of 200, 300, and 400 μL. A mixture of Cremophor^®^ EL and Tween 80 in a 15:85 (*v*/*v*) ratio served as the surfactant (500, 600, and 700 μL), and polyethylene glycol MW200 functioned as the cosurfactant (100, 200, and 300 μL). A 3^3^ factorial experimental design was employed to determine the optimal concentrations of each component. The mixture was homogenized at room temperature for 20 min. A total of 25 mL of distilled water at room temperature was added and stirred at 300 rpm for 15 min.

### 2.5. Characterization of Nanosystems

Nanoemulsions were characterized by dynamic light scattering (DLS) using a Malvern Zetasizer Nano-ZS90 (Worcestershire, UK) to determine the average droplet size and polydispersity index (PDI). A total of 1 mL of nanoemulsion was mixed with 1 mL of deionized water (Simplicity, Merck, Darmstadt, Germany) and filtered through a Milipak-40 0.22 μm filtration unit (Merck, Darmstadt, Germany). The sample was transferred to a disposable polystyrene cuvette (DTS0012) and analyzed using a scattering angle of 173° and 25 °C [15]. The viscosity of the medium was taken as that of water, based on the instrument’s reference values. All measurements were performed in triplicate. Nanoemulsions with a droplet size ≤ 200 nm, monomodal distribution, and a PDI ≤ 0.2 were considered suitable for further study.

### 2.6. Ternary Phase Diagram Construction

SNEDDS formulations were chosen based on the treatments described in Section 2.4 and Section 2.5. Random points were plotted around these formulations to show SNEDDS characteristics. This process helped identify a spontaneous nanoemulsification region within the ternary phase diagram. Each formulation was analyzed by DLS.

### 2.7. Incorporation of ACGs into SNEDDS

Points were randomly selected within the spontaneous nanoemulsification region of SNEDDSs without ACGs to assess how adding the bioactive compound affects the nanodroplets formed. The formulations were characterized using the same methods and criteria previously established.

### 2.8. Thermal Analysis by Differential Scanning Calorimetry (DSC)

For thermal analysis, SNEDDSs loaded with ACGs were evaluated using a differential scanning calorimeter (DSC Mettler Toledo Model 821, Greifensee, Switzerland), equipped with a thermal analysis data station. A total of 5 mg of each sample was placed into an aluminum pan and inserted into the instrument with a temperature increase from 30 to 450 °C at 10 °C/min.

### 2.9. Fourier Transform Infrared (FTIR) Spectroscopy

Using an FTIR spectrophotometer with an attenuated total reflectance (ATR) accessory featuring a diamond tip (Spectro GX FTIR, Perkin Elmer, Waltham, MA, USA), the individual components of the SNEDDSs (MCT, Cremophor^®^ EL, Tween 80 and polyethylene glycol), ACG-enriched fraction, and final SNEDDS formulations were analyzed. Infrared spectra were recorded over 4000–600 cm^−1^ with 24 scans at a resolution of 4 cm^−1^.

### 2.10. SNEDDS Stability Study in Simulated Gastrointestinal System (In Vitro)

An in vitro gastrointestinal tract simulation study was carried out in three stages, studying the oral, gastric, and small intestinal phases, based on previously reported methodologies [16,17] with minor modifications. In the first stage (Table 1), inorganic salts were mixed with porcine mucin (0.3 g/L) to simulate saliva. A 5 mL volume of the SNEDDS was combined with 5 mL of the simulated saliva solution. The pH was adjusted to 6.8 using 0.5 M sodium hydroxide. The sample was incubated for 10 min at 37 °C on an orbital shaker at 100 rpm. An aliquot was taken for nanodroplet characterization. The second phase simulating gastric fluid contained 34.2 mM sodium chloride, 7 mL of hydrochloric acid, and 3.2 g of porcine pepsin.

All components were combined and brought to a total volume of 1 L with water. The pH was adjusted to 2 using either 1 M hydrochloric acid or 1 M sodium hydroxide. Then, 10 mL of simulated gastric juice was added to the previously mixed nanoemulsion and saliva solution; the pH was then adjusted to 2.5 using 1 M sodium hydroxide. The temperature was maintained at 37 °C with continuous agitation at 100 rpm for 2 h. An aliquot of the digested nanoemulsion was collected for evaluation. For the third phase, simulating the small intestine, the pH was adjusted to 7 using 1 M sodium hydroxide. Then, 2.1 mL of bile salts (46 mg/mL) was dissolved in phosphate buffer at pH 7. The pH of the solution was monitored and corrected to 7 as needed with 0.1 M sodium hydroxide. Finally, 1.3 mL of pancreatic lipase (15 mg/mL) was dissolved in 100 mM phosphate buffer at pH 7. The mixture was incubated at 37 °C at 100 rpm for 2 h. During this phase, aliquots of the digested nanoemulsion were collected every 15 min to monitor nanodroplet digestion over 2 h.

### 2.11. In Vitro Release of SNEDDS-ACGs and Korsmeyer–Peppas Release Modeling

Following the methodology reported by Korsmeyer et al. [18] with minor modifications, 10 mL of SNEDDS-ACGs was mixed with 10 mL of a potassium chloride–hydrochloric acid buffer (0.05 M, pH 1.5) and 0.2 mL of pepsin (300 mg/mL). The mixture was stirred for 1 h at 37 °C using a magnetic stirrer (IKA^®^ C-MAG HS10, Wilmington, NC). Subsequently, 4.5 mL of phosphate-buffered saline (PBS, 0.05 M, pH 6.9) and 1 mL of pancreatic α-amylase were added. The sample was stirred at 37 °C and 500 rpm for 3 h. After digestion, the mixture was placed on a cellulose dialysis membrane (Sigma-Aldrich, St. Louis, MO), and the volume was adjusted to 30 mL with PBS (0.05 M, pH 6.9).

Aliquots of 500 µL were withdrawn from the interior of the dialysis membrane at 0, 1, 2, 3, 4, 5, 6 and 7 h. Each aliquot was mixed with 2 mL of Kedde’s reagent, as described in Section 2.3.

The cumulative release of ACGs from the SNEDDS formulation was estimated from the decrease in ACG concentration remaining in the donor compartment according to Equation (1):

(1) $$\mathrm{Cumulative}\mathrm{release}\left(\%\right)=\left(\frac{{\mathrm{TAC}}_{i}-{\mathrm{TAC}}_{t}}{{\mathrm{TAC}}_{i}}\right)\times 100$$

where TAC_i_ is the initial concentration of ACGs in the donor compartment at 0 h, and TAC_t_ is the concentration of ACGs remaining in the donor compartment at time t [6]. Thus, the calculated percentage represents the fraction of ACGs released from the SNEDDS formulation relative to the initial ACG concentration. A mathematical interpretation of ACG data released through DDSolver^®^ was conducted [19], and the release profile was fitted to zero-order, first-order, Higuchi, Hixson–Crowell and Korsmeyer–Peppas kinetic models. The Korsmeyer–Peppas model was used to estimate the release exponent (n) and to characterize the predominant release behavior [18].

### 2.12. Entrapment and Encapsulation Efficiency of SNEDDS-ACGs

Entrapment [20,21] and encapsulation [22] efficiencies were assessed by placing a 10 mL aliquot of SNEDDS-ACGs into a dialysis membrane (Sigma Aldrich, St. Louis, MO, USA), immersed in phosphate-buffered saline (PBS) at pH 7.4. The entire setup was kept on a magnetic stirrer at 500 rpm and 37 °C. Samples of the nanoemulsion were withdrawn from the dialysis membrane after 15 min of stirring, with a second sample collected after 24 h. To quantify the ACG content at each time point, the collected aliquots were homogenized at 5000 rpm for 5 min using a homogenizer (Biospec Model 985370, Xiamen, China). Then, 500 μL of chloroform was added to extract the ACGs from the nanoemulsion. After evaporating the chloroform, the precipitated ACGs were dissolved in 1 mL of methanol. This solution was mixed with 2 mL of Kedde’s reagent, and absorbance was measured at 505 nm using a Jenway 6705 spectrophotometer (Dunmow, UK) [22].

For the encapsulation efficiency calculation, E1 represents the concentration of ACGs associated with the SNEDDS after 15 min of dialysis, whereas E2 represents the concentration of ACGs released after the completion of the 24 h dialysis procedure.

Encapsulation efficiency was calculated using the following Equation (2):

(2) $$\mathrm{Encapsulation}\mathrm{efficiency}\left(\%\right)=\left(\frac{E1-E2}{E1}\right)\times 100$$

For the entrapment efficiency calculation, C1 represents the initial concentration of ACGs in the SNEDDS before dialysis, whereas C2 represents the concentration of ACGs released after 15 min of dialysis. Conversely, entrapment efficiency (Equation (3)) was determined using the conditions outlined above.

(3) $$\mathrm{Entrapment}\mathrm{efficiency}\left(\%\right)=\left(\frac{C1-C2}{C1}\right)\times 100$$

### 2.13. Cell Viability Study Using MC-38 Cell Line

Cell viability was evaluated using murine colon adenocarcinoma cells (MC-38, Kerafast, Inc., Cat. Nr. ENH204-FP, Boston, MA, USA). Briefly, 3 × 10^4^ cells per well were seeded in 24-well plates for each condition, with Dulbecco’s Modified Eagle Medium (DMEM; GIBCO^®^ Cat. Nr. 11995065) supplemented with 10% fetal bovine serum (FBS; GIBCO^®^ Cat. Nr. 26140079), 1% non-essential amino acid solution (Corning^®^ Cat. Nr. 25-025-CI), 10 mM HEPES (Corning^®^ Cat. Nr. 25-060-CI), and 1% penicillin–streptomycin (GIBCO^®^ Cat. Nr. 15240-062). The cells were cultured at 37 °C in a humidified atmosphere composed of 5% CO_2_ and 95% air.

Cytotoxicity was determined from two independent experiments (*n* = 2). Each independent experiment included six technical replicates per condition. The two independent experiments were considered the experimental units for statistical analysis, whereas the technical replicates were used to obtain the response measurements within each experiment. After 48 h, the culture medium was replaced with assay medium consisting of DMEM supplemented with 0.5% (*w*/*v*) bovine serum albumin (BSA; GIBCO^®^ Cat. Nr. 9048-46-8), 1% non-essential amino acid solution, 10 mM HEPES, and 1% penicillin–streptomycin, containing SNEDDS-ACGs at concentrations of 10–60 µg/mL. These concentrations refer to the total concentration of the SNEDDS-ACG formulation, rather than to Kedde-equivalent ACG concentrations.

For comparison, the non-encapsulated ACG-enriched fraction was evaluated under the same cell culture conditions using MCT as the vehicle. The free ACG fraction was tested at concentrations of 10–60 µg/mL. The corresponding vehicle control was included at the highest concentration used in the assay to account for any effect attributable to the vehicle. After 24 h of exposure, cell viability was assessed using the trypan blue exclusion assay in a Neubauer chamber. Cell survival was originally calculated relative to the mean viability of the DMEM control group. The apparent IC_50_ of the complete SNEDDS-ACG formulation was determined by nonlinear regression using log_10_ concentration versus percent survival in GraphPad Prism 6.01.

### 2.14. Statistical Analysis

All statistical analyses, including an analysis of variance and post hoc mean comparisons (Tukey’s test, *p* < 0.05), were performed using Statgraphics Centurion v.16 software (Statpoint Technologies, Inc., Warrenton, VA, USA).

## 3. Results

### 3.1. Acetogenin Profile

The ACG-enriched fraction obtained from the Annona muricata extract used in the present study was previously characterized and identified by the research group that provided the sample [22]. The analysis performed in that study, including extraction, HPLC-DAD, and mass spectrometric characterization, identified a complex mixture of structurally related acetogenins. The main compounds identified included pseudoannonacin, desacetyluvaricin, annonacin, squamostatin-D, isodesacetyluvaricin, squamocin, and bullatacin, along with three unidentified acetogenins [22].

According to the previously reported chromatographic profile, pseudoannonacin was the most abundant compound (60.22%), followed by desacetyluvaricin (8.10%), annonacin (6.17%), three unidentified acetogenins (6.09%, 3.09%, and 3.37%), squamostatin-D (4.36%), isodesacetyluvaricin (3.45%), squamocin (2.99%), and bullatacin (2.16%) [22]. Since the fraction used in the present study corresponded to the previously characterized sample provided by this research group, no additional compound-specific mass spectrometric characterization was performed. Therefore, the ACG composition considered in the present study is based on the previously published analytical characterization of the same ACG fraction [22]. In this respect, bullatacin has demonstrated significant cytotoxicity against colon cancer cells. In one study [23], bullatacin induced apoptosis in SW480 and HT-29 colon cancer cell lines by promoting ATP release and activating macrophages, thereby enhancing phagocytic activity and ultimately leading to cell death.

In other studies, like ref. [24], the annonacin extract showed cytotoxicity, with a median lethal dose (IC_50_) of 0.56 μM in the COR-L23 lung carcinoma cell line. Likewise, annonacin was shown to reduce tumor size in a mouse model at a dose of 0.01 mg/kg [25], underscoring its potential as an antiproliferative agent.

In the case of the MCF-7 breast cancer cell line, significant cytotoxic effects were observed with extracts prepared from different solvents (ethanol, ethyl acetate, and n-hexane fractions), yielding LD_50_ values of 5.3 μg/mL, 2.86 μg/mL, and 3.08 μg/mL, respectively [26]. Additionally, a study on colorectal cancer cells (COLO-205) using ACG extracts reported a median lethal dose of 189.6 μg/mL, derived from ethanolic extracts of soursop leaves [27].

### 3.2. Solubility of ACGs in MCT as Oil Phase

The selection of MCT as the oil phase is related to the structural characteristics of acetogenins (ACGs). These molecules possess a long aliphatic skeleton containing approximately 32–34 carbon atoms, which constitutes a predominantly hydrophobic region, in addition to a terminal lactone group and one or more tetrahydrofuran (THF) rings bearing hydroxyl groups. Although these functional groups introduce polar regions capable of establishing hydrogen-bonding interactions, ACGs retain an extensive hydrocarbon surface that favors their affinity for lipid phases. Therefore, the balance between their hydrophobic and polar regions allows for their incorporation into oil-based vehicles without requiring the molecules to be completely nonpolar [28].

In this context, MCTs, which are mainly composed of medium-chain fatty acid triglycerides (C6–C12), provide a suitable lipophilic environment for the solubilization of hydrophobic compounds. Their ability to solubilize various lipophilic compounds, together with their ease of self-emulsification, has supported their use as the oil phase in SEDDSs/SNEDDSs [29]. The interaction between the hydrocarbon region of ACGs and the acyl chains of MCTs may favor the incorporation of ACGs into the oil phase, while their hydroxyl, ether, and lactone groups may contribute through polar interactions with the ester groups of the triglycerides. Together, these characteristics provide a structural basis for explaining the adequate solubilization of ACGs in MCTs.

Experimentally, ACGs exhibited a solubility of 20 mg/mL in MCT. This solubilization capacity is particularly relevant for SNEDDS development because it allows the required ACG loading to be achieved using a relatively low amount of the oil phase. This may, in turn, contribute to reducing the amount of surfactant required to obtain a formulation with adequate self-nanoemulsification capacity. In addition, the lipophilic nature of ACGs is compatible with the principle of SNEDDSs, which are designed to promote the dispersion and solubilization of compounds with low aqueous solubility [30].

The solubility obtained in MCT is also comparable with some values reported for ACGs in other systems and solvents. In a lipid-based nanosuspension, an ACG solubility of 25 mg/mL in methanol has been reported [6]. Likewise, squamocin and bullatacin were dissolved in dichloromethane at concentrations of approximately 62.3 and 49.8 mg/mL, respectively, for the synthesis of glycosidic conjugates [31]. Petroleum ether has also been used for the extraction and solubilization of ACGs from Annona squamosa seeds, with a reported concentration of 0.91 mg/mL [32]. Although these values correspond to different systems and are not directly comparable because of differences in solvent, composition, and methodology, collectively they support the affinity of ACGs for organic and lipid media. Therefore, the solubility of 20 mg/mL obtained in MCT confirms the ability of this oil phase to incorporate relevant amounts of ACGs and supports its selection for the development of the SNEDDS.

### 3.3. SNEDDS Formulation and Characterization by DLS

After solubilizing the ACG-enriched fraction in MCT, 27 formulations were evaluated using a 3^3^ factorial design. Of the 27 treatments evaluated, 15 met the predefined selection criteria for SNEDDSs, including a droplet size below 200 nm, a PDI below 0.2, and a monomodal size distribution. The 15 selected formulations are presented in Table 2, retaining their original numbering within the experimental design (T1–T9, T12, T15–T18, and T24). As shown, the selected formulations exhibited droplet sizes ranging from 15 to 50 nm and PDI values between 0.04 and 0.18. These characteristics indicate an adequate self-nanoemulsification capacity, and the sizes are below the upper limit of 200 nm commonly considered for SNEDDSs intended for oral administration. The reduction in droplet size can be attributed to the combined action of the surfactant/cosurfactant system, which decreases the interfacial tension between the oil phase and the aqueous medium and promotes the spontaneous formation of nanosized droplets upon dilution. The small droplet size increases the available interfacial area, which may enhance the dispersion and solubilization of highly lipophilic compounds. Therefore, the 15 selected formulations met the established physicochemical criteria for further characterization and evaluation.

### 3.4. Analysis of SNEDDS Droplet Size and Surfactant Effect

Tukey’s mean-comparison tests showed that a higher oil phase content (400 mg) had a significant effect (*p* < 0.05) on droplet size, resulting in larger droplets. In contrast, formulations containing less oil (200 mg) produced smaller droplets, with an average size of 16.03 nm, confirming that the oil phase proportion directly influences the final droplet size, as shown in Table 2. Similarly, surfactant levels (Cremophor EL/Tween 80) had a significant effect on droplet size (*p* < 0.05). Formulations containing higher surfactant amounts (600–700 mg) produced the smallest droplets, highlighting the decisive role of the surfactant fraction in forming finer, more stable nanostructures.

These results are consistent with previous reports indicating that, from a thermodynamic perspective, increasing the oil phase volume increases the volume of the dispersed phase that must be stabilized, thereby increasing the free energy associated with creating new surface area. Under these conditions, the reduction in interfacial tension is insufficient to favor the formation of smaller droplets, resulting in larger droplet diameters. Increasing the surfactant concentration effectively reduces interfacial tension, lowering the energy required to generate new interfaces and facilitating the formation and stabilization of smaller droplets. This energetic balance explains the observed trend toward smaller droplet sizes in systems with higher surfactant content [33].

Lower surface tension helps keep nanoemulsion droplets more evenly dispersed, resulting in a more uniform droplet size distribution. This improves the nanoemulsion’s stability by preventing the formation of larger droplets that tend to settle more quickly. Consequently, surfactants decrease interfacial tension, thereby promoting the formation of nanosized emulsion globules [34]. In this study, MCT was used as the oil phase, as reported in previous research [13,14], yielding particle sizes of 20–40 nm.

### 3.5. Construction of Ternary Diagram

**Figure 1:**
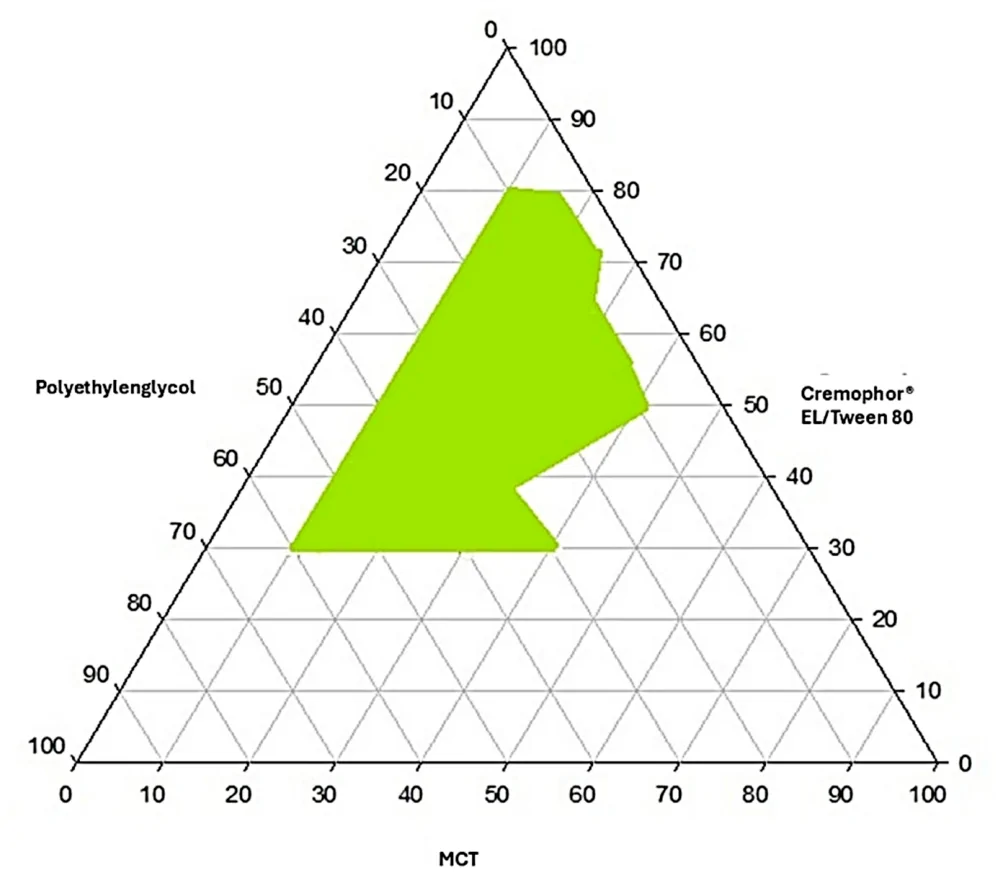
Ternary diagram indicating self-nanoemulsification zone.

The formulations that met the predefined SNEDDS criteria in the 3^3^ factorial design are represented in a ternary phase diagram (Figure 1) using the relative proportions of MCT, the surfactant, and the cosurfactant. These treatments that met the criteria for droplet size (<200 nm), the PDI (<0.2), and monomodal size distribution were used as reference points to identify the region of the formulation space associated with adequate self-nanoemulsification.

Based on this region, an additional exploration of the formulation space was performed by generating point compositions around the selected treatments. These compositions differed from the levels originally established in the 3^3^ factorial design and were used to explore the ternary diagram region in greater detail, where systems with suitable droplet size and PDI characteristics could be obtained. Therefore, these formulations do not correspond to additional treatments of the factorial design but rather to compositions subsequently selected based on the information obtained from that design.

The formulations obtained through this exploration of the formulation space allowed compositions within the self-nanoemulsification region to be identified and those exhibiting suitable physicochemical characteristics for the incorporation of the ACG fraction to be selected.

Instead of testing every possible combination of components, phase diagrams can guide the selection of promising formulations, saving both time and resources. Therefore, choosing the right components for SNEDDS development is essential, especially the oil phase, where the bioactive compound will be solubilized and encapsulated using surfactants. Understanding the role of the lipid vehicle in nanoemulsion development is crucial [35].

In this context, the hydrophilic–lipophilic balance (HLB) is a crucial parameter for assessing the compatibility of nanoemulsion ingredients. Surfactants with high HLB values prefer the aqueous phase, whereas those with low HLB values favor the oil phase.

Surfactants and cosurfactants decrease the interfacial tension between water and oil, forming stable nanoemulsions. This process prevents coalescence and phase separation, ensuring formulation consistency and stability by reducing surface tension [36].

Cremophor^®^ EL, with an HLB of 12–14, and Tween 80, with an HLB of 15, were combined as surfactants. Both exhibit a high affinity for the aqueous phase. Non-ionic surfactants are frequently used in nanoemulsion formulations because of their reduced sensitivity to pH and ionic strength changes, their excellent compatibility with other components, their good physicochemical stability, and their low toxicity [37].

Cosurfactants, like surfactants, play an important role in reducing the surface tension between the oil and water phases. Their properties enhance interactions with surfactant monolayers at interfaces, disrupting the organization and long-range order of surfactant molecules while increasing interfacial fluidity and curvature, which supports the formation of nanoemulsions [38].

The use of Cremophor^®^ EL/Tween 80 as a surfactant and polyethylene glycol as a cosurfactant in research has been documented [39,40].

### 3.6. Preparation of Pseudoternary Phase Diagram

**Figure 2:**
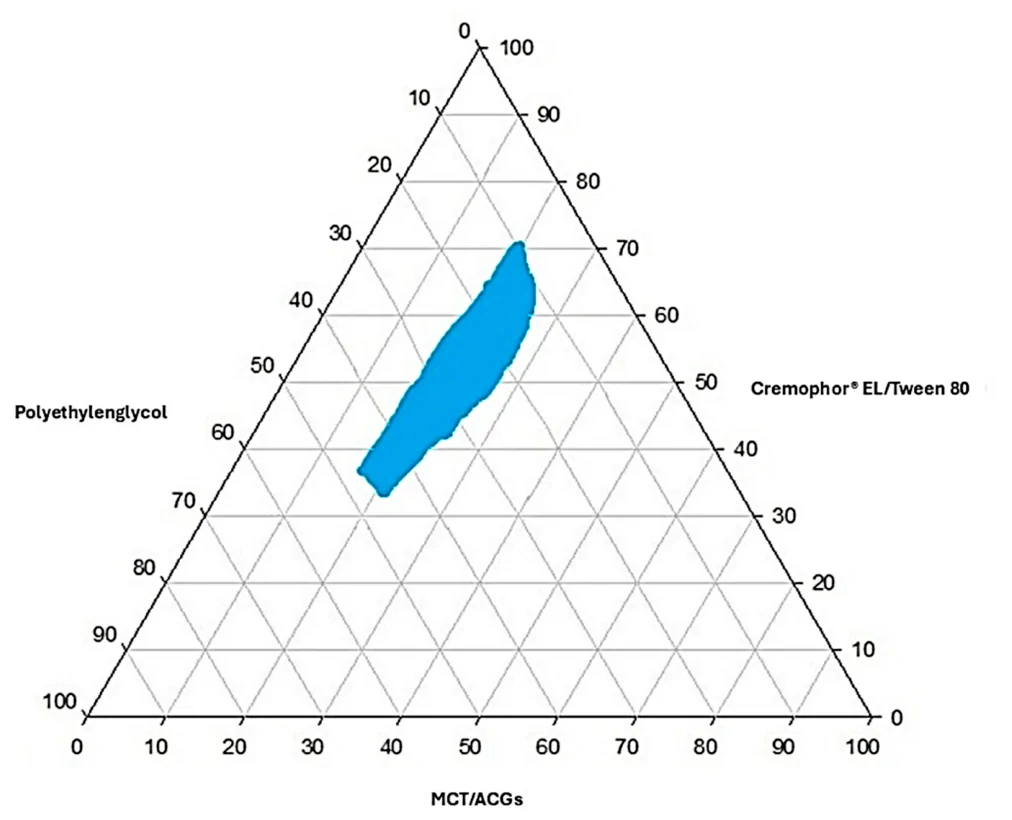
Pseudoternary diagram: auto-nanoemulsification zone can be noticed.

The composition of nanoemulsions with ACGs solubilized in the oil phase is shown in a pseudoternary diagram (Figure 2). The self-nanoemulsifying region is smaller compared to that for drug-free nanoemulsion. There were no statistically significant differences in droplet size or the PDI.

The SNEDDS-ACG nanoemulsions exhibited droplet sizes of 14–40 nm and PDI values of 0.07–0.13 (see Table 3). This behavior could be due to SNEDDSs being specifically designed to solubilize drugs in the nanoemulsion’s oil phase effectively.

The similar droplet sizes and PDI values in Table 2 and Table 3 indicate that the drug is uniformly distributed within the oil phase, without significantly altering the emulsion droplet structure. The phase diagram illustrates the relationship between component concentrations and phase behavior during nanoemulsion formation [41].

A similar study using A. muricata leaf extract with a Cremophor^®^ EL/Tween 80 surfactant mixture reported an optimal surfactant composition of 66%, prepared by sonication to form the emulsion [42]. Predictive modeling software indicated that particles created with Aleurites moluccanus (candlenut) oil as the oil phase and propylene glycol as a cosurfactant would be larger than 400 nm. This is likely due to the long-chain triglycerides (LCTs) in candlenut oil, which tend to increase particle size in emulsions [41]. Our study successfully produced smaller particles and lower PDI values, which could improve absorption via membrane fusion. This enhancement in droplet characteristics may also boost their bioavailability after gastrointestinal uptake [43].

### 3.7. Differential Scanning Calorimetry (DSC) Studies

**Figure 3:**
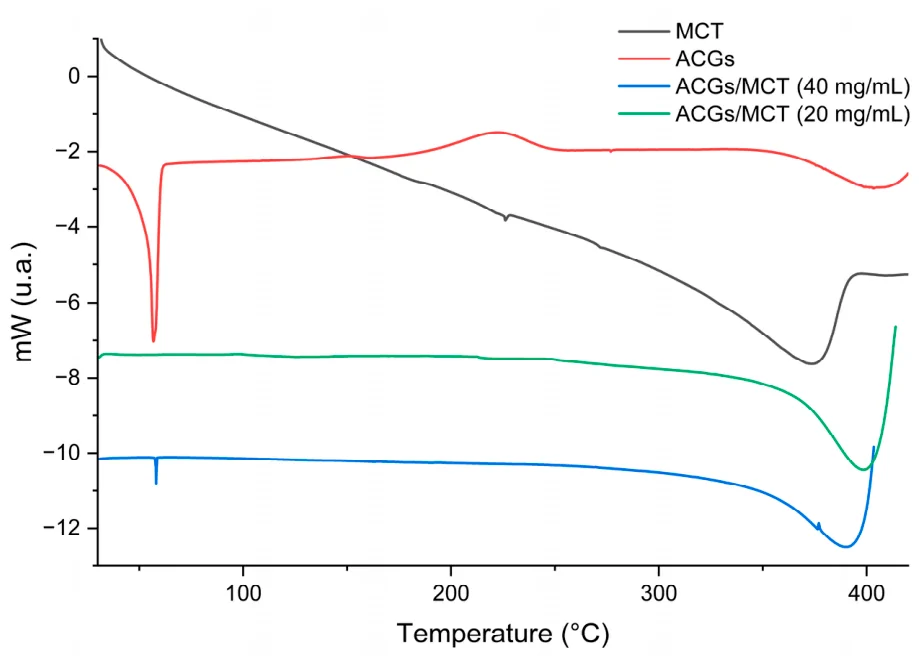
Differential scanning calorimetry (DSC) study of ACGs, MCT and ACG/MCT (20 and 40 mg/mL).

The thermal properties of MCT, the ACG-enriched fraction (20 mg and oversaturated 40 mg), and the MCT–ACG mixture were examined using DSC. At 40 mg/mL, an additional endothermic event was observed at approximately 56.85 °C. This thermal transition was considered in the interpretation of the thermal behavior of the formulation. Because the individual components may exhibit overlapping thermal events, this signal was not assigned exclusively to ACGs. The thermogram for the pure compound showed an endothermic peak at 56.85 °C (Figure 3), corresponding to the melting point of the ACGs and aligning with values reported in earlier studies [44,45].

Conversely, when a solute is fully dissolved in a solvent, as is the case for ACGs in MCT, there is no distinct solid phase of the solute remaining to undergo a phase transition, such as melting. In this state, the ACGs are no longer presented as separate crystalline solids but are fully integrated into a homogeneous molecular mixture. Without a defined solid structure to break down under heating, the DSC analysis did not exhibit a characteristic melting peak.

The thermogram of the MCT–ACG mixture (20 mg) shows no distinct melting peak for the ACGs, which may indicate that the extract is dissolved in the oil phase rather than dispersed as a solid. This is beneficial because it suggests more effective encapsulation, avoiding undissolved precipitates that could reduce the encapsulation efficiency, bioactivity, and stability of the delivery system [30,46].

### 3.8. FTIR Study of Nanoemulsion and Components

**Figure 4:**
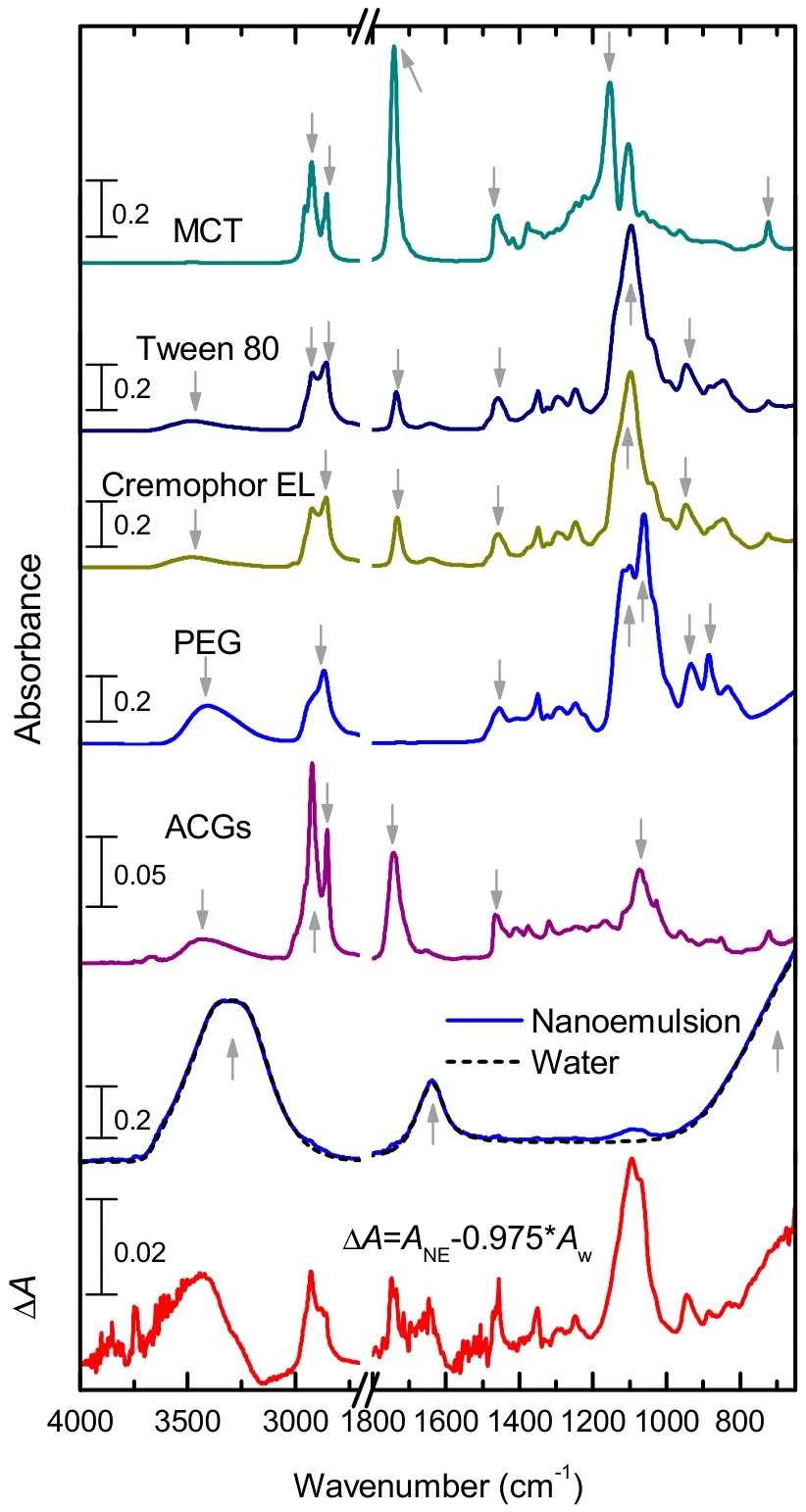
The infrared absorbance spectra of SNEDDS components (MCT, Tween 80, Cremophor EL, PEG), ACGs, the nanoemulsion and water. At the bottom, the difference spectrum of the last two is shown.

Figure 4 displays the spectra from top to bottom for MCT, Tween 80, Cremophor EL, PEG, ACGs, the nanoemulsion, and water, along with the subtraction spectrum of the last two. The absorbance scale of each spectrum is indicated by the bars near the left vertical axis. For clarity, a break in the wavenumber axis between 2700 and 1800 cm^−1^ was added, as no absorption bands occur in that range; note the different scales before and after the break.

The oil phase MCT exhibited molecular vibrations of the main functional groups: bands at 2924 and 2855 cm^−1^ corresponded to C–H stretching vibrations. Carbonyl groups appeared at 1741 and 1154 cm^−1^. Bending vibrations of saturated fatty acids were observed at 1458 and 722 cm^−1^, while bands between 950 and 800 cm^−1^ indicated C–H bond deformations. For Tween 80 and Cremophor EL^®^, similar bands were found around 3460 cm^−1^ (O–H stretching), 2920–2860 cm^−1^ (C–H stretching), and near 1735 cm^−1^ (C=O stretching).

Additionally, a band at 1096 cm^−1^ was associated with C–O vibrations. In the case of PEG, a more intense and asymmetric band of stretching O-H vibrations was observed, with a maximum at 3410 cm^−1^; a sharp C–H band appeared around 2867 cm^−1^, with a shoulder at higher wavenumbers; C=O bands appeared close to 1742 cm^−1^; and a doublet at 1100 and 1060 cm^−1^ indicated C–O stretching.

For ACGs, a smaller absorbance scale is observed because the sample was in powdered form and the contact with the diamond ATR crystal is limited. However, the absorption bands characteristic of ACGs are well-defined.

A broad band centered around 3440 cm^−1^ indicates O–H stretching vibrations. Sharp bands at 2922 and 2853 cm^−1^ correspond to C-H stretching modes. A strong peak near 1742 cm^−1^ identifies carbonyl (C=O) groups, which are common in ketones or esters in some ACGs. Additionally, signals in the 1000 to 1300 cm^−1^ range are associated with C–O bonds.

In the spectrum of nanoemulsions, the high water content causes water’s vibration modes to dominate the spectrum: a broad band centered at 3315 cm^−1^ corresponds to -OH stretching modes; the bending of H-O-H molecules appears as the band at 1638 cm^−1^, while the broad band at the lowest wavenumbers is due to water’s vibration modes.

However, some differences between the absorbance spectra of the nanoemulsion (A_NE_) and water (A_w_) are noticed mainly in the 1400–1000 cm^−1^ range, which can be enhanced with the subtraction ∆*A* = *A_NE_* − 0.975 ∗ *A_w_*, where the factor 0.975 accounts for the large water content and is close to the volume fraction in the nanoemulsion.

As can be noticed in the bottommost spectrum in Figure 4, ∆*A* shows a peak at 3420 cm^−1^, and the shift from that of water at 3315 cm^−1^ indicates the rearrangement of the O-H bonds of water molecules interacting with the hydrophilic groups around the droplets; the sharp band at 2920 cm^−1^ and the shoulder at 2860 cm^−1^ of the C-H stretching modes of all organic compounds present in the nanoemulsion; 1740–1745 cm^−1^ of C=O for MCT, Cremophor EL, Tween 80, and ACGs; 1460 cm^−1^ for all organic compounds; 1353, 1249, and 945 cm^−1^ for Cremophor EL, Tween 80, and PEG; 1094 and 1070 cm^−1^ for all organic compounds; and 885 cm^−1^ for PEG. The FTIR spectra showed changes in the O–H stretching region following the formation of the nanoemulsion. These changes may reflect modifications in the local molecular environment of hydroxyl-containing and other hydrophilic components upon interaction with the aqueous phase. The observed spectral behavior is consistent with changes in hydration and hydrogen-bonding interactions within the nanoemulsion; however, FTIR spectroscopy alone does not allow these effects to be unequivocally distinguished or to establish specific hydrogen bonds between water and individual formulation components. Therefore, the FTIR results are interpreted as supportive evidence of changes in the molecular environment of the hydrophilic domains of the SNEDDS rather than definitive evidence of specific hydrogen bond formation.

### 3.9. In Vitro Stability Studies

**Figure 5:**
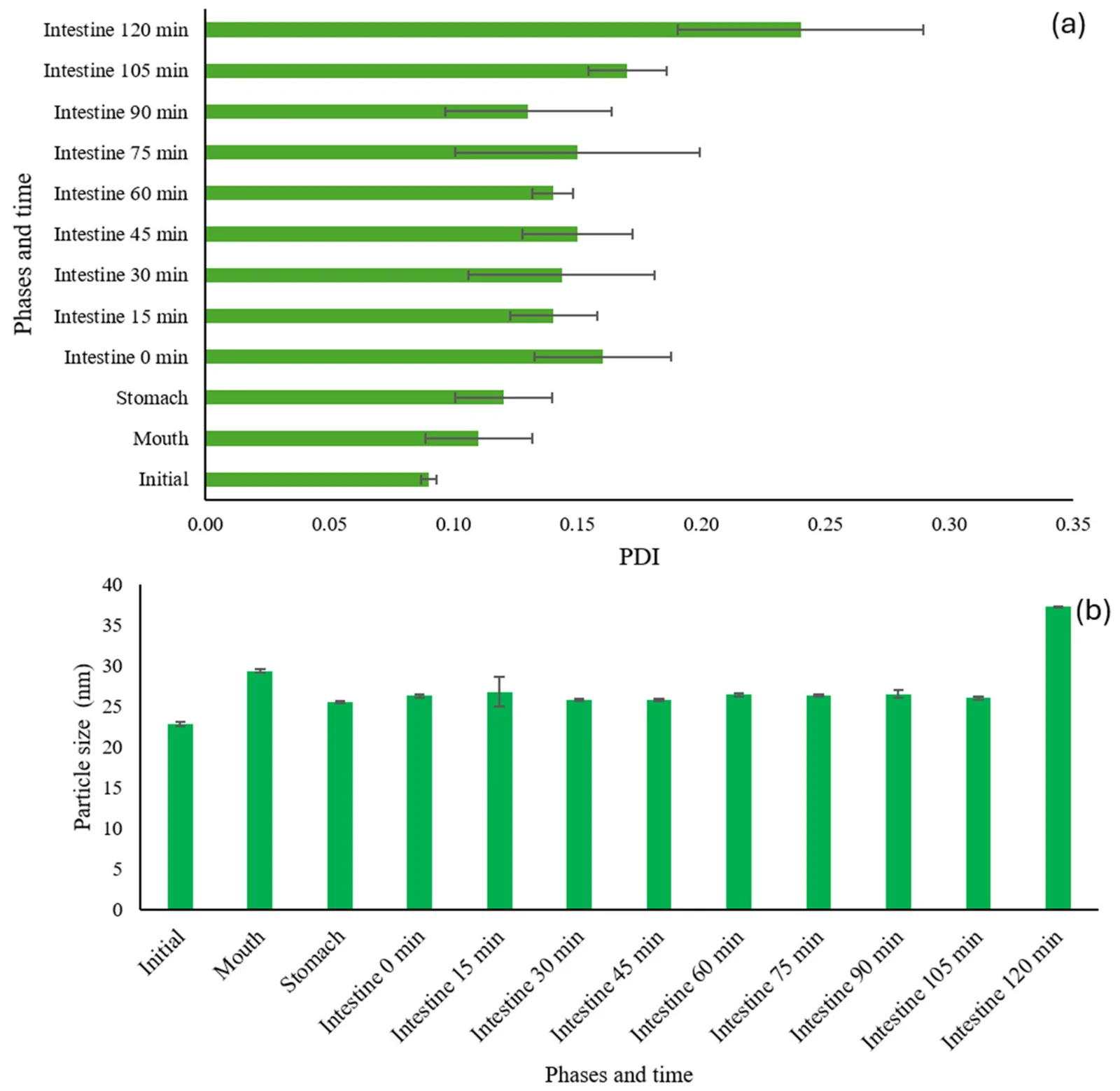
Particle size and polydispersity index of SNEDDS-ACGs during in vitro digestion study. (**a**) Polydispersity index. (**b**) Droplet size.

In vitro assessments of ACG-loaded nanoemulsions that aimed to evaluate their stability within a simulated gastrointestinal tract (GIT) were performed. Initially, nanoemulsions with ACGs (SNEDDS–ACGs) had a droplet size of 21.95 nm and a PDI of 0.1. Figure 5a displays the PDI values across various GIT simulation phases, while Figure 5b illustrates the droplet sizes recorded during each stage of the in vitro study.

In the initial (oral) phase, the nanosystem showed no notable changes in droplet size or the PDI, suggesting that the nanoemulsion remained stable under simulated salivary conditions. This phase includes salts and mucin, which could potentially destabilize emulsions via flocculation [47].

During the second phase (gastric), the environment became acidic due to the gastric secretions involved in digestion, including enzymes. The chemical stability of the characteristic functional groups of ACGs should also be considered during exposure to acidic release conditions. The ACGs incorporated into the SNEDDS are highly lipophilic and are solubilized within the MCT-based lipid phase, which may provide a protective microenvironment and reduce their direct exposure to the aqueous acidic medium. This acidity can compromise nanoparticle integrity, potentially leading to coalescence or flocculation, thereby increasing droplet size [48]. The potential susceptibility of ACGs to acidic conditions is an important consideration because these compounds contain a γ-lactone moiety and other oxygen-containing functional groups. Nevertheless, in the present formulation, ACGs were incorporated into the lipid/surfactant matrix of the SNEDDS rather than being directly exposed as free compounds to the aqueous acidic medium. Upon dispersion in gastrointestinal fluids, SNEDDSs spontaneously form a fine oil-in-water nanoemulsion, providing a lipophilic microenvironment that can reduce the direct interaction of the encapsulated compounds with the external aqueous phase. Thus, the formulation may provide a protective effect against unfavorable gastrointestinal conditions and contribute to preserving lipophilic bioactive compounds during gastric transit. In the present study, the maintenance of the nanoscale characteristics of the formulation during the simulated gastrointestinal process supports its physicochemical stability under the conditions evaluated. Nonetheless, no significant changes in the PDI were observed, most likely because no degradative components were present that could harm the nanoparticle structure. Droplet size also remained stable, likely due to the absence of enzymatic degradation, as proteolytic enzymes lacked a suitable substrate. Additionally, the non-ionic surfactants used (Tween 80/Cremophor^®^ EL and polyethylene glycol) are less vulnerable to environmental changes, thereby maintaining emulsion stability.

In the final phase, the nanoemulsions are exposed to alkaline digestive fluids containing bile salts, pancreatic lipases, ions, and other components, which facilitate the breakdown of substances before their absorption in the small intestine. These fluids change the pH from acidic to neutral or slightly basic. Previous research using Cremophor^®^ EL as a surfactant demonstrated stability across a pH range of 6.4 to 7.4 [49].

Additionally, these digestive fluids act as biosurfactants in physiological processes, playing a key role in digestion and nutrient absorption. They may interact with or compete with the original surfactants on the nanoparticle surface; however, in lipid-interfaced regions, stability primarily depends on the primary lipid, its interactions and packing at the interface, and its interactions with bile salts. This interaction affects the extent to which bile salts are absorbed into lipid films, thereby preparing the interface for lipolysis [50].

In our study, we monitored droplet size every 15 min during the intestinal phase simulation. No significant changes were seen in the oral and gastric phases until minute 120 of the intestinal phase, when a marked increase occurred. During the first 105 min of the intestinal phase, particle size stayed between 25.42 and 26.01 nm (Figure 5b). However, at minute 120, it suddenly rose to 37.29 nm. However, because drug release, permeability and intestinal absorption were not directly evaluated in this study, these results cannot be used to establish a specific absorption pathway [50].

The increase in droplet size observed during the intestinal phase, from approximately 25–26 nm to 37–38 nm after 120 min, accompanied by an increase in the PDI, may be attributed to changes in the composition and organization of the droplet interface during digestion. In the intestinal environment, bile salts act as biosurfactants and can interact with the surfactants initially present at the oil–water interface, promoting their displacement or reorganization and modifying the interfacial properties of the droplets [51]. These changes may affect colloidal stability and promote some degree of aggregation or coalescence, resulting in an increase in droplet size and polydispersity. Similar changes in droplet size have been associated with coalescence and interfacial reorganization processes during the gastrointestinal digestion of nanoemulsions [52].

On the other hand, although the surfactants used in the formulation are not the main substrates for pancreatic lipases, MCTs are composed of triacylglycerols that can be hydrolyzed during the intestinal phase. The action of pancreatic lipase on triacylglycerols mainly generates free fatty acids (FFAs) and monoacylglycerols (MAGs), which exhibit interfacial activity and can modify the composition of the layer surrounding the droplets [53]. Subsequently, bile salts promote the solubilization of these lipolysis products through the formation of mixed structures, including mixed micelles composed of bile salts, FFA, and MAG. Therefore, the observed increase in droplet size and the PDI can be interpreted as the combined result of lipid hydrolysis, the incorporation and redistribution of lipolysis products at the interface, interactions with bile salts, and the possible partial aggregation or coalescence of the droplets [54]. Since these processes were not individually evaluated in the present study, the observed changes should be considered as an overall response of the nanoemulsion to the intestinal digestion conditions.

Similar studies with Gelucire^®^ 44/14 showed that this excipient can inhibit the digestion of superficial micelles in such nanosystems, likely due to its low concentration [55]. As a non-ionic surfactant, Gelucire^®^ 44/14 exhibits lower enzymatic activity compared to pancreatic lipases, and its non-ionic nature probably prevents full enzymatic action.

### 3.10. In Vitro Release Study of SNEDDS–ACGs and Modeling Using Korsmeyer–Peppas Model

**Figure 6:**
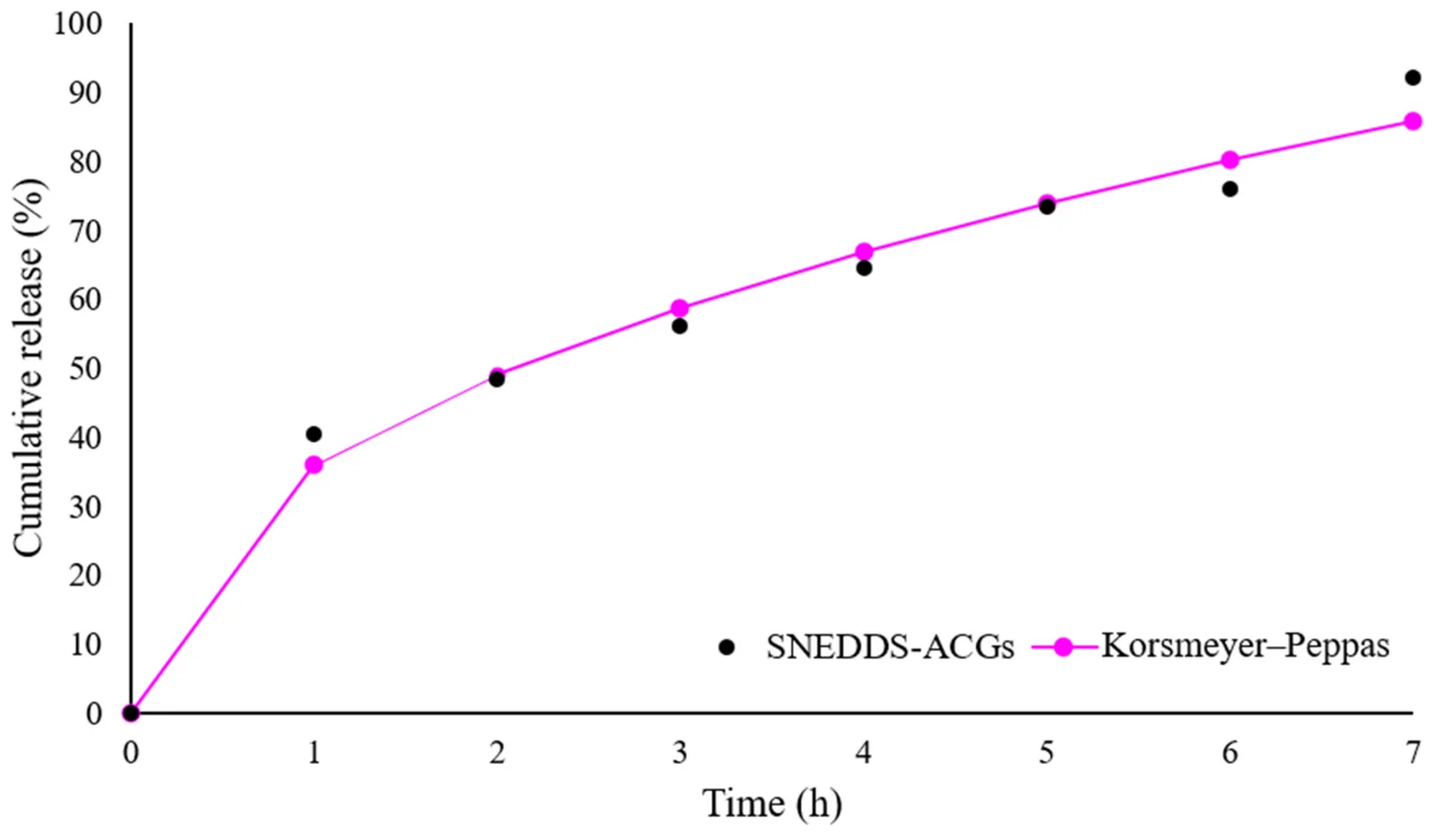
Cumulative release of ACGs from SNEDDS and Korsmeyer–Peppas model.

As shown in Figure 6, the cumulative release profile of ACGs was monitored over 7 h at 1 h intervals. The cumulative release increased from 40.45% at 1 h to 48.88%, 56.16%, 64.56%, 73.33% and 76.07% at 2, 3, 4, 5, and 6 h, respectively, reaching 92.20% at 7 h. Thus, the majority of the ACG fraction quantified by the Kedde assay was released from the SNEDDS formulation during the first 7 h under the experimental conditions evaluated. This profile is consistent with previous studies on SNEDDSs containing bioactive compounds, which have reported release periods of approximately 8 h [56,57]. To investigate the release behavior, the experimental profile was fitted to five kinetic models: zero-order, first-order, Higuchi, Hixson–Crowell, and Korsmeyer–Peppas. The corresponding R^2^ values were 0.2235, 0.8132, 0.9429, 0.7167, and 0.9524, respectively. Among the evaluated models, Higuchi and Korsmeyer–Peppas provided the highest coefficients of determination, indicating that diffusion-related processes may contribute substantially to ACG release. The Korsmeyer–Peppas model showed a slightly better fit based on R^2^ and additionally provided the release exponent (*n*), which can be used to obtain information about the predominant release mechanism.

For the Korsmeyer–Peppas model, the release exponent was approximately k = 5.75, n = 0.45. Values close to the Fickian range have been associated with diffusion-controlled release in spherical systems, whereas higher values may indicate contributions from other transport mechanisms [40]. Therefore, the obtained “n” value suggests that diffusion may be the predominant mechanism governing ACG release, although a contribution from other release processes cannot be excluded. This interpretation is consistent with a previous SNEDDS study using similar excipients, including Cremophor EL and Tween 80, and a lipophilic drug such as tamoxifen, in which Fickian diffusion was reported [58].

Overall, the gradual release profile and the relatively high fit obtained with the Higuchi and Korsmeyer–Peppas models suggest that diffusion plays an important role in the release of ACG from the SNEDDS. The Korsmeyer–Peppas model is particularly useful in this context because the release exponent provides additional information for interpreting the transport mechanism [59]. However, given the similarity in the fit of the Higuchi and Korsmeyer–Peppas models, the release process should be considered predominantly diffusion-associated rather than attributed exclusively to a single mechanism.

### 3.11. Entrapment and Encapsulation Efficiency of SNEDDS–ACGs

The high entrapment efficiency (96%) and encapsulation efficiency (92.59%) indicate that most of the initially incorporated ACGs were associated with and retained within the SNEDDS formulation under the respective experimental conditions. Given the high lipophilicity of ACGs and their solubilization in the MCT-based lipid phase, these values primarily reflect the incorporation and retention of ACGs within the lipid-based formulation rather than physical entrapment within the defined lipid-based system. Because ACGs are highly lipophilic and are incorporated into the oil phase of the SNEDDS, the terms entrapment efficiency and encapsulation efficiency were defined operationally according to the analytical procedures used in this study. Entrapment efficiency (EE) was calculated as the percentage of the initial ACG amount associated with the lipid-based formulation after the removal of the non-associated fraction. Entrapment efficiency measures the percentage of ACGs that bind to or remain on the SNEDDS formulation, while encapsulation efficiency reflects the proportion of ACG solubilized within the lipid phase of the SNEDDS and its retention after aqueous dispersion [44]. SNEDDS–ACGs exhibited an entrapment efficiency of 96% and an encapsulation efficiency of 92.59%. These results indicate the effective incorporation and retention of the ACG fraction within the lipid-based SNEDDS formulation under the conditions evaluated. Because SNEDDSs form dynamic oil-in-water nanoemulsions upon aqueous dispersion, the observed retention is more appropriately attributed to the solubilization of ACGs within the lipid phase and their association with the dispersed formulation rather than to localization within a discrete lipid-based system. Most ACGs were located within the dispersed formulation. This likely results from the solubilization of ACGs in the oily phase, which promotes their localization within the inner compartment.

### 3.12. In Vitro Cell Viability Study in MC-38 Cell Line

**Figure 7:**
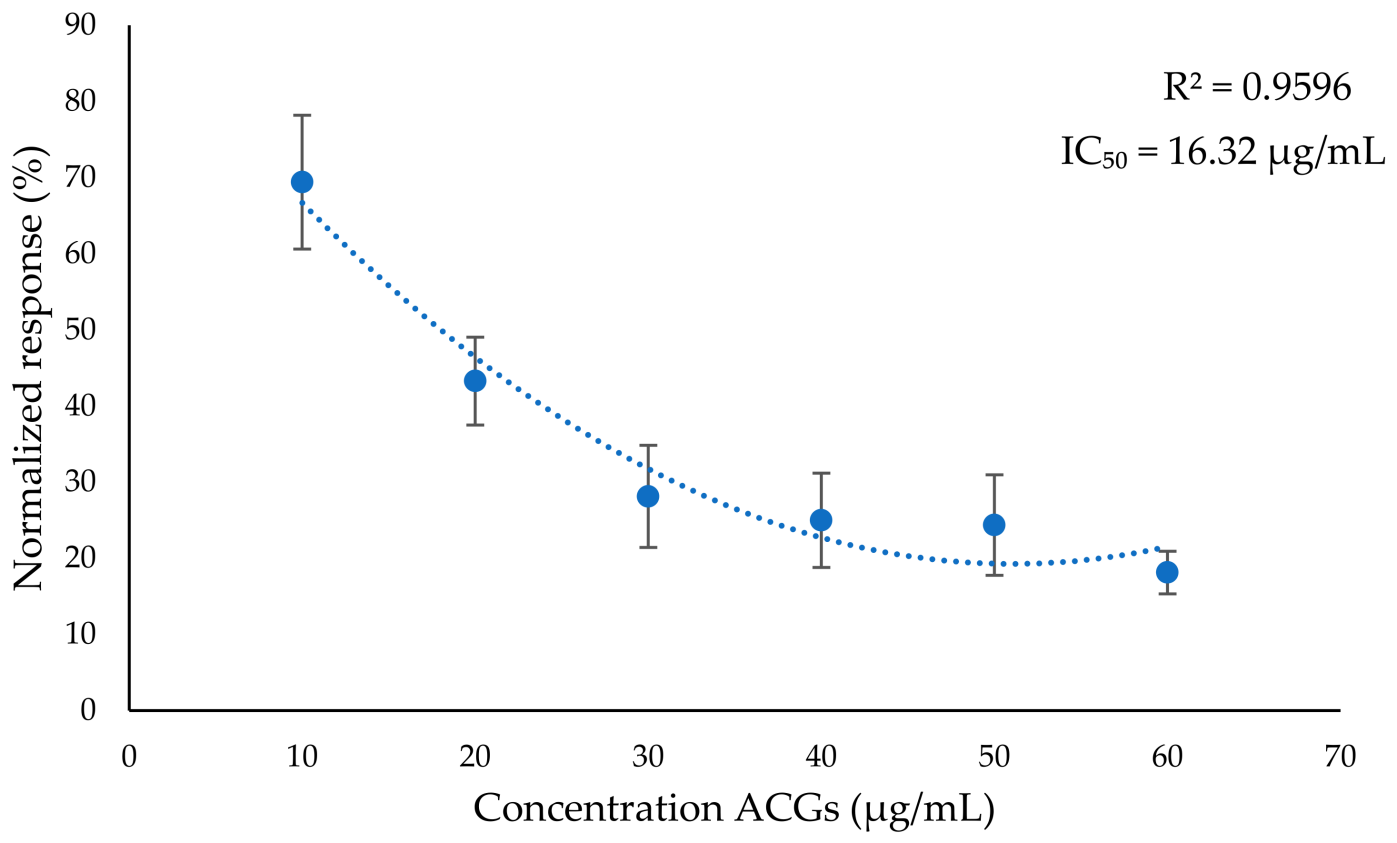
MC38 cell response to different concentrations of ACGs. Results are presented as mean ± standard deviation.

**Figure 8:**
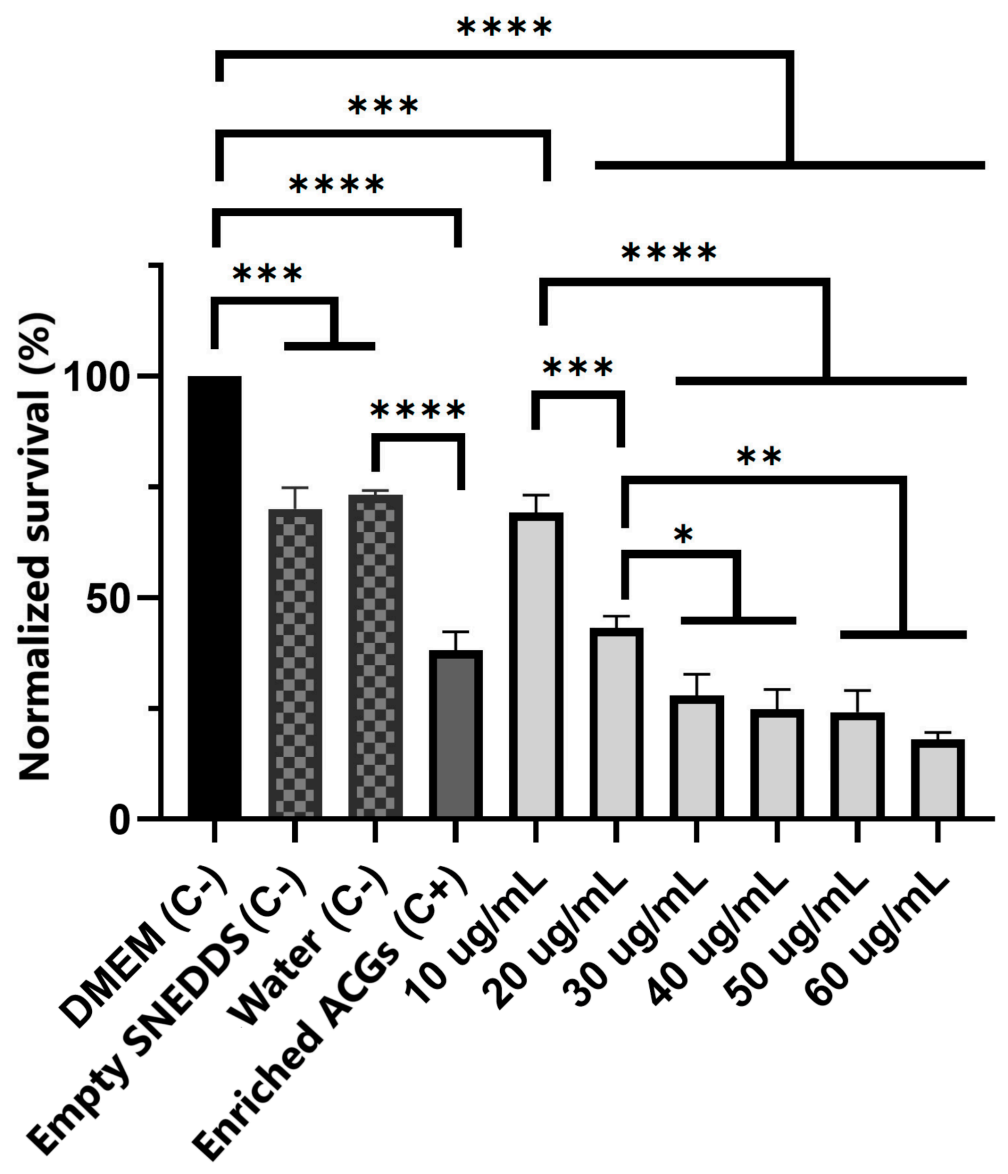
Cell survival rate in MC-38 colon cancer cells treated with SNEDDS-ACGs. Statistical analysis was performed using one-way ANOVA followed by Tukey’s post hoc test. Results are expressed as mean ± standard deviation (*n* = 2). * *p* < 0.05; ** *p* < 0.01; *** *p* < 0.001; **** *p* < 0.0001. C−: negative control; C+: positive control (non-encapsulated ACG fraction, 383 µg/mL).

The evaluation of cell viability showed that the ACG-loaded nanoemulsion exhibited cytotoxic activity against the murine colon adenocarcinoma cell line MC-38, with an IC_50_ of 16.32 μg/mL (Figure 7). For comparison, the non-encapsulated ACG exhibited an IC_50_ of 383 μg/mL, under the experimental conditions described above. This value represents the response of the free ACG-enriched fraction under the specified assay condition and should not be interpreted as the IC_50_ of an individual acetogenin. Likewise, concentrations of 30, 40, 50, and 60 μg/mL showed a progressive decrease in cell viability (Figure 8), consistent with a concentration-dependent effect. The cytotoxicity assay showed that SNEDDS-ACGs reduced MC-38 cell survival in a concentration-dependent manner, with an apparent IC_50_ of 16.32 μg/mL when survival was normalized to the DMEM control. However, the empty SNEDDS also produced a reduction in cell survival, indicating that the formulation vehicle contributed to the overall cellular response. Notably, at 10 µg/mL, SNEDDS-ACGs produced a survival level close to that observed with the empty SNEDDS control. Therefore, the observed cytotoxicity cannot be attributed exclusively to the ACG fraction, and the IC_50_ of 16.32 μg/mL should be interpreted as an apparent IC_50_ of the complete formulation rather than as an ACG-specific IC_50_.

The concentrations used in the cytotoxicity assay represent the concentrations of the complete SNEDDS-ACG formulation and were not expressed as Kedde-equivalent ACG concentrations. The Kedde assay was used for the analytical quantification of the total ACG content of the formulation. Accordingly, the present results provide evidence of the cellular effect of the complete ACG-loaded SNEDDS formulation, while the specific contribution of ACGs relative to the vehicle requires further controlled evaluation.

However, this result should be interpreted considering that the evaluation was performed only in this cell model and that further studies are required to determine the mechanisms responsible for this effect and to establish the selectivity of the formulation.

ACGs exhibit recognized antitumor activity, although the molecular mechanisms involved depend on the specific acetogenin, cell type, and experimental conditions. One of the proposed mechanisms involves the inhibition of respiratory Complex I [24]. The inhibition of NADH dehydrogenase can impair oxidative phosphorylation [60], decrease ATP production, and promote processes associated with cell-cycle arrest and apoptosis.

In this context, some acetogenins present in *Annona muricata*, such as squamocin and bullatacin, have been reported to interfere with cell proliferation and promote apoptosis in different cancer models. For example, squamocin has been reported to induce cell-cycle arrest at the G1 phase, increase caspase-3 activity, and promote PARP cleavage, events associated with the activation of apoptosis [31]. Likewise, different cellular stress signals can activate the extrinsic and intrinsic apoptotic pathways through caspase-8 and caspase-9, respectively, with the involvement of signaling pathways including Akt, ERK1/2, p38, and JNK [61]. Alteration in mitochondrial function can also lead to the loss of membrane potential, decreased ATP production, and increased oxidative stress.

Increased levels of reactive oxygen species (ROS) can promote the oxidation of lipids, proteins, and nucleic acids and contribute to mitochondrial dysfunction [62]. In addition, some acetogenins have been reported to modulate apoptosis-associated proteins, such as Bax and Bak; activate caspase-3; and interfere with processes related to DNA replication and repair, including the inhibition of topoisomerase I [62]. However, because these molecular mechanisms were not directly evaluated in the present study, they should be considered possible explanations for the observed activity rather than experimentally demonstrated mechanisms.

The observed cytotoxic activity can also be contextualized with previous studies of nanostructured systems based on *Annona muricata*. Nanoparticles have been developed using different materials, including gold [63], silver [64,65,66,67,68], zinc [69,70], and solid lipid nanoparticles [71], using extracts from different parts of the plant. Some of these systems have shown cytotoxic activity against different cancer cell lines (Table 4). However, several of these studies used cell lines other than MC-38 and, in some cases, human models of different cancer types. Therefore, these results are presented only as bibliographic context and should not be considered direct potency comparisons.

Some studies using *Annona muricata* extracts have also employed dimethyl sulfoxide (DMSO) as a vehicle, a solvent capable of modifying cell membrane fluidity and permeability and facilitating the uptake of certain molecules. In the present study, DMSO was not used because the ACG fraction was directly incorporated into the SNEDDS. Importantly, the empty SNEDDS produced a modest decrease in cell viability, indicating that the vehicle may partially contribute to the observed response. This response is consistent with the potential interaction of surfactants and other formulation components with cell membranes when present at sufficiently high concentrations. Therefore, the inclusion of the empty SNEDDS as a vehicle control allowed us to partially distinguish the response associated with the ACG-loaded formulation from that produced by the vehicle itself. However, the experimental design does not allow the individual contribution of each excipient to be determined.

The selection of an oil phase based on MCT may also facilitate the incorporation of highly lipophilic compounds. For example, MCT-based systems have been reported for the oral delivery of hydrophobic compounds to improve solubilization and release, including valsartan [72] and antiplatelet agents [73]. Similarly, nanoemulsion systems have been developed for plant extracts with limited aqueous solubility and high lipophilicity [74]. These findings support the use of lipid-based vehicles as a strategy for incorporating hydrophobic bioactive compounds, although differences among the systems prevent establishing a direct relationship between these reports and the cytotoxic activity observed in the present study.

An important consideration is that the cytotoxicity of SNEDDS-ACG was evaluated only in MC-38 cells. Therefore, the results demonstrate the cytotoxic activity of the formulation against this specific cell model but do not establish its selectivity toward cancer cells or its biocompatibility. Future evaluation in nontumorigenic intestinal or colonic cells will allow the relative selectivity of the formulation to be determined and will contribute to establishing its safety profile. Accordingly, the present results should be interpreted as evidence of cytotoxic activity against MC-38 cells rather than as evidence of antitumor selectivity or therapeutic efficacy.

Another limitation of the present study is that not all chromatographic constituents present in the ACG fraction could be structurally assigned. Although several acetogenins have previously been identified in Annona muricata and some constituents could be tentatively associated with previously reported profiles, their individual structures were not confirmed by high-resolution mass spectrometry (HRMS) or tandem mass spectrometry (MS/MS) in the present study. Therefore, the observed biological activity should be attributed to the ACG fraction as a whole rather than to a specific acetogenin. Comprehensive molecular characterization using HRMS/MS will be necessary in future studies to establish the composition of the fraction more precisely and to relate its constituents to the observed biological activity.

As an additional reference, previously reported IC_50_ values for 5-fluorouracil (5-FU) against MC-38 cells show considerable variability depending on the experimental conditions. For example, one study reported an IC_50_ of 0.22 µM after 48 h, whereas another reported a value close to 5 µM using an MTT assay [75,76]. These differences illustrate that IC_50_ values obtained under different exposure times, viability assays, and experimental conditions should not be interpreted as direct measures of relative cytotoxic potency. Accordingly, the IC_50_ value of 16.32 μg/mL obtained for SNEDDS-ACG in the present study is interpreted primarily as evidence of cytotoxic activity under the specific experimental conditions used, rather than as a direct potency comparison with 5-FU.

## 4. Conclusions

The results obtained demonstrate that SNEDDS-ACG represents a nanosystem with potential to improve the incorporation and activity of acetogenins from Annona muricata. The medium-chain triglyceride (MCT)-based oil phase showed an adequate capacity to solubilize the ACG fraction and facilitate its incorporation into the system. DSC analysis supported the solubilization of ACG in the oil phase, as no evidence of a crystalline phase attributable to the incorporated compounds was observed.

The combination of surfactants and cosurfactants exhibited a high self-nanoemulsification capacity, resulting in systems with droplet sizes below 50 nm and favorable polydispersity values. The encapsulation efficiency reached 92.59%, indicating a high incorporation of the ACG fraction into the system. The FTIR analysis showed that the final nanoemulsion retained spectral features associated with the carbonyl-containing components of the formulation. The absorption band observed in the region of approximately 1740–1745 cm^−1^ cannot be assigned exclusively to ACGs because similar carbonyl absorptions were also observed for MCT, Tween 80, and Cremophor EL. Therefore, the FTIR results are interpreted as evidence of the presence of carbonyl-containing functionalities within the formulation rather than as compound-specific evidence for ACGs. The absence of new spectral bands suggests that no evident chemical changes were associated with the incorporation of the ACG fraction. During simulated in vitro gastrointestinal digestion, the formulation maintained nanoscale droplet characteristics throughout the oral and gastric phases and most of the intestinal phase. An increase in droplet size and the PDI was observed after 120 min of the intestinal phase, indicating changes in the physicochemical characteristics of the formulation under the simulated intestinal conditions. Because the digestion study evaluated droplet size and the PDI rather than ACG release or intestinal absorption, these results should be interpreted as evidence of physicochemical behavior during simulated digestion rather than as evidence of compound release or a specific absorption pathway.

The SNEDDS-ACG formulation also showed cytotoxic activity against murine colon adenocarcinoma MC-38 cells, with an IC_50_ of 16.32 μg/mL, whereas the non-encapsulated ACG fraction exhibited an IC_50_ of 383 µg/mL. These results indicate a higher apparent cytotoxic activity of the ACG fraction when incorporated into the nanosystem. Although the evaluation was performed in a specific cell model, the results provide evidence of the biological potential of the formulation against MC-38 cells. Further studies incorporating additional cell models, reference pharmacological controls, and a more detailed molecular characterization of the ACG fraction will contribute to a broader understanding of its activity, selectivity, and mechanisms of action.

## Figures and Tables

**Table 1:** The composition of the simulated gastrointestinal solution used in in vitro digestion studies.

Compound	Formula	Concentration (mM)
Ammonium nitrate	NH_4_NO_3_	4.1
Sodium chloride	NaCl	27
Potassium chloride	KCl	2.7
Potassium citrate	C_6_H_5_K_3_O_7_	1.0
Potassium phosphate	KH_2_PO_4_	4.1
Urea	CO(NH_2_)_2_	3.3
Calcium chloride	CaCl_2_	1.3

**Table 2:** SNEDDS composition. Droplet size and PDI.

Treatment	MCT (μL)	Cremophor^®^/Tween 80 (μL)	PEG (μL)	Droplet Size (nm)	PDI
T1	200	500	100	17.01 ± 0.16	0.11 ± 0.02
T2	200	500	200	17.33 ± 0.73	0.09 ± 0.01
T3	200	500	300	19.53 ± 1.13	0.11 ± 0.04
T4	200	600	100	16.25 ± 1.27	0.11 ± 0.01
T5	200	600	200	18.65 ± 1.41	0.07 ± 0.02
T6	200	600	300	16.67 ± 0.34	0.05 ± 0.01
T7	200	700	100	15.81 ± 0.38	0.05 ± 0.01
T8	200	700	200	16.03 ± 0.28	0.05 ± 0.01
T9	200	700	300	16.11 ± 0.66	0.04 ± 0.01
T12	300	500	300	32.60 ± 2.24	0.15 ± 0.02
T15	300	600	300	26.96 ± 1.24	0.12 ± 0.02
T16	300	700	100	21.48 ± 0.92	0.14 ± 0.02
T17	300	700	200	26.13 ± 3.11	0.18 ± 0.03
T18	300	700	300	24.72 ± 0.95	0.16 ± 0.05
T24	400	600	300	50.18 ± 1.91	0.17 ± 0.04

The reported values correspond to the mean ± SD (*n* = 3).

**Table 3:** SNEDDS formulations incorporating acetogenins and their DLS characterization.

MCT (mg)	Cremophor^®^ EL/Tween 80 (mg)	PEG (mg)	Droplet Size (nm)	PDI	Concentration of ACGs (mg/mL)
150	700	150	14.64 ± 0.44	0.10 ± 0.01	3.00
140	640	230	15.57 ± 0.89	0.08 ± 0.02	2.80
230	600	170	19.12 ± 0.55	0.10 ± 0.01	4.60
130	540	330	16.01 ± 0.35	0.07 ± 0.01	2.60
230	490	280	21.95 ± 0.96	0.10 ± 0.01	4.60
150	400	450	21.07 ± 0.24	0.09 ± 0.02	3.00
200	350	450	40.23 ± 9.73	0.13 ± 0.02	4.00

The reported values correspond to the mean ± SD (*n* = 3).

**Table 4:** Reported cytotoxicity of *Annona muricata*-based systems in different cancer cell models.

Nanoparticle	Cell Line	IC_50_
Gold nanoparticles [63].	MCF-7	500 μg/mL
Silver nanoparticles [64,65,66,67,68].	A-549 PC-3 HeLa PNT1A AMJ-13 HCT116 HCT-116	6 μg/mL 48.17 μg/mL (fruit) 47.58 μg/mL (leaf) 38.58 μg/mL (fruit) 57.63 μg/mL (leaf) 375.68 μg/mL (fruit) 112.29 μg/mL (leaf) 17.34 μg/mL 8.5 µg/mL 2.004 µg/mL
Zinc nanoparticles [69,70].	MOLT4 A549 HCT116	60.13 μg/mL 96.67 μg/Ml 68.5 μg/mL
Lipid-based solid nanoparticles [71].	MCF-7	12 μg/mL

## Data Availability

The data that support the findings of this study are openly available in Zenodo at https://doi.org/10.5281/zenodo.19099175.
